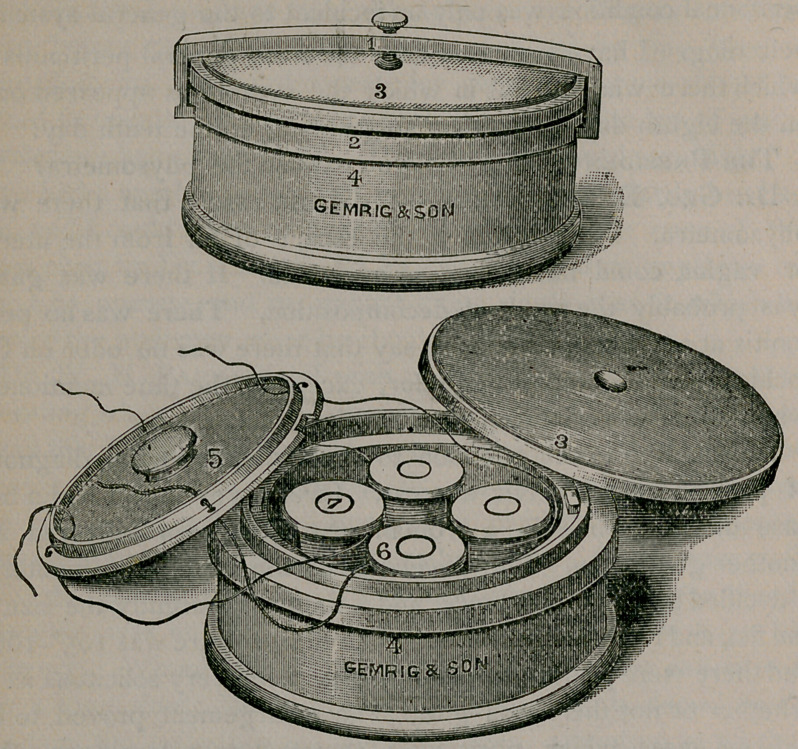# Philadelphia Obstetrical Society

**Published:** 1889-09

**Authors:** J. M. Baldy

**Affiliations:** Secretary; 328 South Seventeenth Street


### PHILADELPHIA OBSTETRICAL SOCIETY.

FRIDAY, June 7, 1889.

The president, Dr. Theophilus Parvin, in the chair.

Dr. Wm. Goodell reported the following history of a case of extra-uterine fœtation, and exhibited the specimen :—

The patient had been married for a number of years without conceiving. Her catamenia had been regular up to the time when they ceased for nearly seven weeks and morning sickness set in. The next monthly period was free for a few hours and then merely a show of blood which lasted several days. During this dribble severe intercostal pains, lasting two hours, followed a movement of the bowels. For several days there was great soreness of all the muscles. At regular intervals these intercostal pains reappeared and were always followed by much muscular soreness. There were few pelvic pains, nothing like cramps, and Dr. Goodell was called in on account of a continuous dribble of blood which had lasted for three weeks. During this metrostaxis membranes were twice passed, which were supposed to be fragments of an early miscarriage. Dr. Goodell found an irregular tumor on the left of the womb, closely adhering to it and pushing the fundus over to the right.

In view of the history, a diagnosis of extra-uterine fœtation was made, and the operation was promptly performed three months after the cessation of the last regular monthly period. There was no appearance of old or of fresh blood in the abdominal cavity, such as is usual in many of these cases when rupture has taken place. But of course blood escaped during the breaking up of numerous adhesions to the rectum and the broad ligament. The specimen shows the left ovary and the corresponding tube greatly enlarged by a deposit of placental tissue. Dr. Osler, who was kind enough to examine the specimen for me, states that the chorion villi are unmistakably present. No fœtus was discovered, but it may have perished and became absorbed, or it is possible that it may have escaped into the abdominal cavity through an opening made accidentally into the sac, during the process of enucleation. So vascular was the sac that a stream of blood spurted out from this tear as if it came from a large vessel. Apart from a nervous attack of vomiting, which lasted nearly twenty-four hours, the convalescence was interrupted.

DISCUSSION.

Dr. J. PRICE.—I am satisfied that Dr. Goodell's explanation of the absence of the fœtus is correct. I could cite two or three cases and an experience of my own which supports this view. Mr. Tait's first two cases made tedious recoveries, and in both he failed to find the fœtus. Some time ago I did a section with a doubtful diagnosis. Some one standing by asked me what I expected to find. I replied, "One of twelve things." I went on and removed a large adherent tube, ruptured, with abdomen pretty well filled with clot. I then irrigated the abdomen. After using one pitcher, the water turned perfectly clear. To make the toilet thoroughly satisfactory, I used the second pitcher of water, and, in finishing the second toilet, washed out a little boy. In this case I am satisfied that the peritoneum could have taken care of the fœtus by digestion, as probably occurred in Mr. Tait's cases.

Dr. HOWARD A. KELLY.—I think that this case illustrates how readily we can make a satisfactory diagnosis, given symptoms being present. With a certain order of symptoms and signs we can with the utmost certainty diagnose extra-uterine pregnancy in a certain proportion of cases. In another large proportion of cases, it is a matter of mere conjecture, until the abdomen is opened. I operated this week on such a problematical case, one of the two possibilities being extra-uterine pregnancy. Such proved to be the condition, although no fœtus was found. I found the sac and the placenta within the ruptured tube.

In a recent book on this subject by Strahan, he unfortunately fails to notice some experiments on the disappearance of the fœtus after its expulsion into the abdominal cavity. Leopold has experimented by introducing fœtuses into the abdominal cavity of dogs. These have been digested, until the period of the more distinct development of the bony tissues has been reached. After that period they have become sources of irritation and have been cast off by suppuration.

Dr. M. PRICE.—I do not think that a study of the cases on record will make a man perfectly satisfied that he can say when he has a case of extra-uterine pregnancy. The ablest men throughout the world have satisfactorily decided that question. They have made mistakes time and time again. They have cut for supposed extra-uterine pregnancy and found something else. They have cut for something else and found extra-uterine pregnancy. It is very difficult to decide until the abdomen is opened.

A ruptured extra-uterine pregnancy can only develop in the broad ligament. If it ruptures into the peritoneum, there is not a single case on record where, if the operation is delayed a number of days, that the fœtus has not disappeared. Hundreds of cases are on record. I have, myself, seen eight or ten where the fœtus could not be found, where the microscope positively showed the presence of extra-uterine pregnancy. Those cases that go on to development are those in which there has been first a rupture into the broad ligament, and then development up to a certain time when the child can resist the digestive action of the peritoneum.

I would ask if this woman or any other woman with extra-uterine pregnancy could be benefited by electrical treatment. There is no question in my mind that in these cases electricity has done a vast deal of harm and has aggravated symptoms already existing and has imperilled the woman's life to a greater extent than if she had been left entirely to nature. The knife, as Dr. Goodell has used it, is the only treatment. Delay is not justifiable at any period, unless when the case comes into the hands of the surgeon the child has passed to that degree of development that warrants its being left to the period of viability. All of these cases demand operative procedure at an early period, if they come into the hands of the operator.

DR. WILLIAM GOODELL.—I fully agree with the remarks of those gentlemen who hold to the uselessness of electricity. I think that there is only a single class of extra-uterine fœtation in which electricity might be valuable, and that is in the early weeks before hemorrhages have occurred. An examination of the specimen before us shows, to my mind, that hemorrhages must have taken place in the tube, forming layers of organized clot. In such cases I do not see how it is possible for electricity to do anything but harm. In those occasional rare specimens in which the chorion has remained intact, resembling an abortion coming away without rupture, the ovum being nothing more than a delicate, but shaggy bladder, with the fœtus inside, I can understand how electricity can do good by destroying the life of the fœtus. Then everything might readily become absorbed; but as we can never know positively beforehand whether or not hemorrhage has occurred, my own feeliegs are in favor of immediate section.

While the difficulties of diagnosis are undoubtedly very great, this need not interfere with our treatment. We find a woman suffering from certain pelvic symptoms and we discover an extra-uterine tumor of some kind. Now a painful pelvic tumor must be removed, whatever it is. The only change in the treatment would be to hasten on the operation were the symptoms pointing in the direction of extra-uterine fœtation.

DR. GEORGE E. SHOEMAKER reported a case of "Puerperal Septicæmia." Operation not indicated. Autopsy.

Because of the interest which attaches at the present time to the question of the place of laparotomy in the treatment of post-puerperal trouble, this case is reported. It is one in which the question of operative interference was weighed and decided in the negative—correctly, as was shown by the autopsy.

B., aged 26 years, Irish, having had one child with indefinite history of after-trouble at that time, was delivered April 23, of a small male child before full term.

At five and one-half months she had a free hemorrhage from the vagina, which was stopped under another practitioner's care. Three days prior to the labor, while asleep in bed at 3:30 A. M., she was again seized with free bleeding. She sent for another practitioner, who gave ergot, and the hemorrhage ceased. There was no pain, and the ergot did not bring on labor. Just forty-eight hours later, while again asleep in bed, another free hemorrhage occurred, again without pain. Several hours later, or two days after the first hemorrhage, the writer was called.

The bed-clothing, body linen, and mattress were much soiled by large quantities of dried blood. The vagina contained considerable offensive clot, the os was dilated to the size of a half-dollar, and was occupied by clot, the pains were absent, the child living, and presenting L. O. A. The margin of the placenta was easily felt posteriorly and to the right, so that, as might be expected from the history, there was partial *placenta prævia*.

Efforts to remove the septic surroundings were begun at once. The nurse in attendance, ignorant and unclean, with a suppurating skin eruption in the palm of the hand, was discharged, and a trained nurse obtained from that admirable charity, the Visiting Nurse Society. Soiled clothing was removed, a vaginal bichloride of mercury douche given, the matted pubic hair cut off, and the patient's body and hips thoroughly bathed in 1 to 1,000 sublimate solution, this being a greater strength than would have been used but for the decomposition. All this was several hours before the labor terminated; and from a time at least four hours prior to delivery, strict antisepsis was maintained by the free use of mercurials on hands and about the patient. It was too late; the decomposed blood had poisoned the system before delivery—a clear case of ante-partum infection. Hemorrhage did not recur, so that no measures were necessary for its arrest after the writer assumed charge of the case. It was the intention to perform version at once on its recurrence, as much blood had been lost and the child was weak. The labor terminated spontaneously without complications, the child being alive and the mother in fair condition, though with apparently some rise of temperature. Unfortunately the thermometer was not used. A 1 to 4,000 bichloride of mercury hot intra-uterine injection was given immediately, but the temperature rose above 103° eight hours later; that is, too soon for post-partum infection under the circumstances. There was, however, no tenderness over the uterus, no pain, no sign of peritonitis, and no abnormal odor to the lochia, which were apparently normal. Another 1 to 4,000 bichloride injection was, however, carried into the uterus, and the treatment with quinine and whiskey begun, which lasted throughout the case. Epsom salts, dr. ss, hourly till the bowels moved, disturbed the stomach without improving the general condition later.

There was no decided change for four days; the fever reached about 103° in the afternoon, but the stomach acted well and the strength was fairly maintained. The lochia were without abnormal odor and of fairly natural appearance till the end of the second day, when the injections were stopped, to be resumed later on the appearance of slight odor. There was at no time any great tenderness about the uterus or over the abdomen, which remained soft and undistended. As the case progressed, no definite local complications appeared. There was no uræmia and no sign of mercurial poisoning; the bowels acted well. On one occasion a considerable swelling occupied the abdomen in the median line below the umbilicus. At first glance it was supposed to be the uterus distended by clot, but when light pressure by the hand was made upon it, much to the writer's surprise the swelling at once and permanently disappeared, while at the same time there was an audible escape of gas, probably from the vagina.

From the patient's mental condition, exact information was not obtainable, and there was no opportunity to repeat the experience. Was this physometra? The pelvis was repeatedly examined, but no accumulations could be felt. There was evidently no considerable amount of necrotic material in the uterus, for the discharges did not indicate it. There were no symptoms of peritonitis at any time, and in spite of the fixed determination of the patient to die, the outlook was fair until after the fourth day. From this time till the eighth and final day the progress was downward. The temperature became 104° in the afternoons, with violent active delirium, obstinate insomnia, and profuse perspiration, especially at night. The stomach and intestines remained in fair order, but the nervous condition was very bad.

The temperature rose, on the morning of the eighth and final day, to 106.6°, and the patient died of exhaustion. No attempt at laparotomy was made, because none seemed indicated in the absence of peritonitis or any sign of definite pelvic trouble. With the utmost difficulty, permission was obtained to make an autopsy, the husband being present to see that nothing was removed.

Autopsy, twenty hours after death; rigor mortis; emaciation; abdomen very slightly distended. Abdominal cavity contained the usual amount of serum, which was pinkish red, not turbid; contained no flocculi, but simply stained muslin without residue. No sign of lymph exudate or pus. Peritoneum and intestines pale and smooth without any adhesions and without hemorrhagic spots. Intestines not over-distended. The uterus, as large as a large fist, was distended by gas, and when compressed remained collapsed like a bag. It was pale bluish white in color, incision showing the walls to be about one-third of an inch in thickness; the cavity empty of all fluids. A pinkish red, thin, transparent coating of mucus covered the lining membrane. This mucus was not abundant enough to flow—simply a coating. It was examined under the microscope and found to contain no pus, but to be made up largely of epithelial cells of various ages, showing very marked fatty change. The large amount of fat was remarkable. It was not extraneous, as no lubricant could be obtained for use on the hands. The left tube was of the size of the little-finger, and when first touched apparently contained gas like the uterus. It collapsed with handling, but no liquid could be found in the uterine cavity which could have been squeezed from it. It was not adherent, and when delivered with the ovary through the abdominal incision and incised did not look unhealthy, and contained no fluid. The right tube was smaller, and contained no gas. It was adherent to the uterus and the right side of the pelvis by old adhesions, but was delivered with the ovary through the abdominal incision, incised, and found empty. Both ovaries were of normal size, the tubes of a bluish pink, and pale like the other organs and the peritoneum from loss of blood. No collection of pus or any source of infection, removable or otherwise, could be found in the abdomen or pelvis. Other portions of the body were not examined, owing to the exigencies of the *post mortem*. There had, however, been nothing to call attention to them. The cause of death then was general septicæmia, with no continued source of infection near the point of original departure.

The question of the treatment of post-puerperal septic conditions by abdominal section, as suggested and practiced by Mr. Tait and others, is one of great importance. The reports of successful cases demonstrate beyond doubt that in laparotomy a new and very valuable means of combating some forms of a fatal disorder has been developed.

Just now we are in need of more definite clinical knowledge as to when the belly should and when it should not be opened. Where peritonitis persists, the profession is rapidly reaching the conclusion that laparotomy increases the patient's chances, or if there is a distinct purulent collection, that it affords almost her only chance.

Whether pus is to be found in the peritoneum, in the tubes, or in the connective tissue, whose intercellular spaces are continuous with the lymphatic vessels, its removal, followed by drainage, is unquestionably indicated. Where the uterus itself is infected to such a degree that the local use of dull curette and antiseptic douche will not remove the source of infection, there is nothing suggested which is more promising than hysterectomy, though in practice this is likely to prove disappointing from prior general infection. It must not be forgotten, however, in these days of readiness for operation, that there are cases, such as that here reported, where there is early a general infection, not from pus, for there is none formed, nor yet from any remaining focus of infected material; and where it would be just as useless to incise the peritoneum or remove tubes as to remove the stomach hours after a poisonous dose of atropia. There are some cases in which, if they go on long enough, abscess may form late, especially from emboli, where phlebitis exists; but the pus may lie in lung or brain, as well as in more accessible localities, and the hope of re covery does not lie in laparotomy.

What is demanded therefore in each case is a careful study of that case by itself, and repeated examination for foci of infected material. When these are found, they should be removed, if accessible, at the earliest possible moment, and by any safe means which will thoroughly do the work.

### DISCUSSION.

DR. WILLIAM H. PARRISH.—The question of laparotomy for septic infection accompanying or following labor is one of great importance. Doubtless many errors have been made in not opening the abdomen, while against this is the fact that at the present stage of abdominal surgery, we should be on our guard that we do not go to the other extreme and open the abdomen in cases of septic infection following labor, when the operation is not indicated. It has so happened that in an acquaintance with the Philadelphia hospital, extending over fifteen years, I have seen a goodly number of autopsies in cases of septic infection after labor. I have seen few instances in which the autopsy showed that laparotomy would have been of any special value. Where there has been a pus accumulation without fatal general septic infection preceding it, and that pus cavity is so located that that it can be opened and drained or entirely removed, the operation is a proper one. If there is reason to believe that there is an accumulation of pus in the peritoneal cavity, it will be right to open the peritoneal cavity and remove the pus, provided the woman is not in a moribund condition. There are, however, cases in which the purulent peritonitis develops very late in the history of the case. There are not a few instances in which the septic infection, as it has extended from the uterus, shows its local effects in the lymphatics, and the fatal result is probably determined before the peritonitis takes on a very active form. I am sure that I have seen this occur. In an endemic in which twenty or thirty autopsies were made, we found the peritoneum in various stages of inflammation. Cases that died early showed inflammation of the lymphatics, and the formation of pus in the lymphatics, particularly of the broad ligament and uterus, with swelling of the areolar tissue and degeneration of the peritoneum. In other cases, the condition was more advanced, with the formation of lymph and flocculi and turbid fluid in the peritoneum. In other cases, where the patient lived still longer, we found a larger quantity of purulent looking fluid. I believe that if these cases had been operated on, the result would not have been favorably influenced. In fact, I think that in some the fatal result would have been promoted by operation. It requires more judgment, and I think probably more skill, to determine when the abdomen should be opened in these cases than to do the operation.

I have in a few instances opened the abdomen after labor, and, as I think, have thereby saved the patient. It has so happened that in all the cases in which I have opened the abdomen after labor the pus has been in the areolar tissue of the pelvis. These cases have recovered. I have not operated on a patient after labor who has not recovered. I do not recall a single instance of pus in the tubes. There was a limited quantity of thickened fluid, but nothing like pyosalpinx. I think that this is rare, especially in endemic septic infection.

DR. J. M. BALDY.—I agree with Dr. Parrish in regard to the difficulty of deciding which of these puerperal cases are subjects for operation and which should be let alone. At one time I thought that it was rather easy to distinguish; but as cases came one after another into my hands, I found it extremely puzzling to know what to say. If there is pus in the tube, which I found in one case, it is easy to settle the question. It is often difficult to say whether or not there is pus at all. In the vast majority of cases in which I have been asked to decide for or against operation, I have advised waiting, and all of these cases have recovered, showing that there was no pus. If you can make up your mind positively that there is pus or purulent fluid, there would be no question as to the advisability of operating. I should not wait because the woman was far gone, in the hopes of bringing her up. I think that the pus is at the bottom of the trouble, and that the only way of saving her is to stop its absorption at once, by removal. The great difficulty is to decide whether pus be present or not, and it requires caution, or we shall be led into many operations which will be unnecessary.

I think in the case of Dr. Shoemaker, that the question of operation would not have come up at all. It was not a case of peritonitis, nor were there symptoms of local trouble. From the report I can see no indications for the use of the knife. I think that this was clearly a case of absorption of ptomaines, and in such a case there would never be formation of pus.

Dr. M. PRICE.—I have had rather an unfortunate experience with this operation. In three cases that I have had there has been persistent vomiting. For days there had been fever and quickened pulse and a well-marked chill. Upon examination there was unquestionable evidences of pelvic inflammation. In one case tubal trouble was well-marked, and the uterus could be mapped out from the tubes. In this case the operation was performed on the eleventh day, after it had been determined that the woman had peritonitis. Three pints of pus poured out. The pus had burrowed up behind the kidneys on either side. The case was fatal.

The second case was one of criminal abortion, where the girl fell into Dr. Musser's hands at the last minute, and he sent her to me. Within six hours I operated and found three pints of pus. No well-marked tubal trouble could be found. All the surroundings were in a semi-gangrenous condition. The patient died.

The third case was seen a few weeks ago, the pelvis was as solid as if it had been frozen. She had a chill and the broken tea-leaf appearance of the vomit. She finally consented to an operation, and on opening her from one to two pints of pus escaped. Nothing was done but to open the belly, break up the inflammatory adhesions, wash out the cavity, and use drainage. In these cases, I think that early operative procedure would have given the patients a chance for their lives If I were to-night to see a case of septic peritonitis where there had been a chill, some distention of the bowel, a fixed condition of the uterus, I should not hesitate longer than to obtain my instruments.

Dr. J. PRICE.—Dr. Parrish has well selected his cases; he has not made any mistake; he has operated in suitable cases, and others he has permitted to die because any operative interference would simply have hastened death. In all cases in which we have operated, we have been able to place our fingers on something before operating. The cases reported by my brother were all dying. It is unfortunate for surgery that we should be forced to operate on a dying patient, A large number of puerperal cases have been saved. Dr. Baldy has saved two cases. I have had a number in my own practice. Dr. Bernardy has had one, and I could cite a number of other cases. In none of the cases cited by Dr. Parrish did he put his hand on anything on which to operate, and he cannot cite a single case in which he opened the abdomen when he should not have done so.

Dr. W. H. PARRISH.—I wish to add one word in regard to operating when the conditions seem to be fatal. I did not mean to say that I would not operate on an abscess, believing such to be present, when the patient is very ill. In one instance, I removed one and a half gallons of pus from a patient in a condition of extreme emaciation and almost ready to die. She is now well. I should hold off from operating in a case in which the blood-poisoning was so great that there was no possible hope for recovery. Where there is an encysted abscess, the patient will live a long time; but in epidemic diseases, with dense septic infection, the patients, even when first seen, are often so ill that exploratory incision would certainly not be a proper thing to do, inasmuch as it would add to the mortality following surgical operations and deter others from operating and other patients from being operated upon. If there is reason to believe that there is a pus cavity, I should operate if the patient was almost *in extremis*. Where, on the other hand, the blood-poisoning was the main trouble, I should not open the abdomen as a matter of exploration.

Dr. JOSEPH HOFFMAN.—There is one point in connection with the case of Dr. Shoemaker to which I would call attention, and that is the use of the bichloride and the absence of odor. It seems to me that the absence of odor must have been due to the bichloride. The statement that there was no odor is perhaps a little too wide, inasmuch as disinfection was used persistently. It seems very evident that the case was one of general septicæmia from the preceding dirt, and that the peritoneal condition was only an incident to the general systemic poisoning. I have seen one case die from general peritonitis in which there was pus, but in which the symptoms appeared only on the eighth day, the patient succumbing on the tenth day.

THE PRESIDENT.--How do you explain the physometra?

DR. GEO. E. SHOEMAKER.—I did not assert that there was physometra. I only asked if the escape of air from the uterus or vagina could have been so explained. If there was gas it was probably the result of decomposition, There was no peritonitis at any time. When I say that there was no odor on the lochia, I mean no abnormal odor, except at the time mentioned, when there were indications for disinfection.

I would like to call attention to one difficulty in the diagnosis of post-puerperal pelvic abscess. Over a year ago I had a bad case of septicæmia in a woman who had been delivered by another gentleman. She developed on the left side of the uterus a decided sense of resistance, and a tumor apparently the size of the fist, and tender on pressure. The temperature was 105°-106°, and there were profuse sweats at night. I felt very solicitous as to whether or not there was pus. The enlargement proved to be a fæcal mass, which purgatives removed in a few days. No operation was performed, and to-day there is not a healthier woman in the city.

DR. H. M. WEEKS exhibited "An Antiseptic Ligature Box."

This box is presented to the profession for preserving and carrying ligatures that have been prepared and rendered aseptic or antiseptic, enabling the operator to cut his ligatures and suture, at the time of operating, without danger of soiling or infecting the portion not required for immediate use. It is made of a fine quality of earthenware, thus securing strength and durability; at the same time it is light, compact, ornamental; and last, but not least, it can be furnished at a price that will enable every one practicing surgery to provide himself with one or more. The box can be had in any color desired, or with any decoration the consumer may wish.

The accompanying cut represents the different parts as follows:

The box is round, four inches in diameter and two inches high, with an outside cover; No. 2, that is held in position by a neat clamp; No. 1, which, when adjusted, is prevented from slipping by a slot on either side of the hand or flange at the top of the box, the screw holding the cover tightly down upon the rubber washer; No. 3, which encircles the top, and renders the box absolutely air and fluid tight, so that the ligatures can be carried constantly in any solution desired without danger of leakage.

The inner cover, No. 4, is a flat disk with a slot cut in the edge to allow it to be placed in position, and held by two small catches placed on opposite sides of the box; the small knob in the center serves to turn and place and remove the cover. There are four holes perforating this cover for the four sizes of silk generally used, and half an inch from the edge of the cover there is a raised band, also perforated for the silk to pass, thus making it impossible for the end of the ligature to drop back into the box when cut. This cover rests upon a ledge, and is left in place, except when necessary to fill the reels or spools with silk, or the box with solution.

The reels or spools, No. 6, four in number, stand upright, and are held in position by separate spindles, No. 7. The whole box is highly glazed; there is no metal nor anything that can be acted upon by any solutions, and the material from which it is made can be subjected to any amount of heat, either dry or boiling. It can be taken apart in a very few seconds, and every part thoroughly cleansed.

Should any of the parts break, they can be replaced, as they are interchangeable.

They may be obtained from J. H. Gemrig & Son, 109 South Eighth street, Philadelphia.

J. M. BALDY, *Secretary.*

328 SOUTH SEVENTEENTH STREET.

## Figures and Tables

**Figure f1:**